# Generation of an Alagille syndrome (ALGS) patient-derived induced pluripotent stem cell line (TRNDi032-A) carrying a heterozygous mutation (p.Cys682Leufs*7) in the *JAG1* gene

**DOI:** 10.1016/j.scr.2023.103231

**Published:** 2023-10-18

**Authors:** Omer Hatim, Ivan Pavlinov, Miao Xu, Kaari Linask, Jeanette Beers, Chengyu Liu, Karsten Baumgärtel, Melissa Gilbert, Nancy Spinner, Catherine Chen, Jizhong Zou, Wei Zheng

**Affiliations:** a National Center for Advancing Translational Sciences, National Institutes of Health, Bethesda, MD, USA; b iPSC Core, National Heart, Lung and Blood Institute, National Institutes of Health, Bethesda, MD, USA; c Transgenic Core, National Heart, Lung and Blood Institute, National Institutes of Health, Bethesda, MD, USA; d Travere Therapeutics, 3611 Valley Centre Drive, Suite 300, San Diego, CA, USA; e Division of Genomic Diagnostics, Department of Pathology and Laboratory Medicine, The Children’s Hospital of Philadelphia, Philadelphia, PA, USA

**Keywords:** Alagille syndrome, ALGS, Human induced pluripotent stem cells, iPSC culture, iPSC passaging, iPSC characterization

## Abstract

Alagille syndrome (ALGS) is an autosomal dominant, multisystemic disorder due to haploinsufficiency in either the *JAG1* gene (ALGS type 1) or the *NOTCH2* gene (ALGS type 2). The disease has been difficult to diagnose and treat due to its muti-system clinical presentation, variable expressivity, and prenatal onset for some of the features. The generation of this iPSC line (**TRNDi032-A)** carrying a heterozygous mutation, p.Cys682Leufs*7 (c.2044dup), in the *JAG1* gene provides a means of studying the disease and developing novel therapeutics towards patient treatment.

## 1. Resource table

**Table:** 

Unique stem cell line identifier	TRNDi032-A
Alternative name(s) of stem cell line	**HT977A; NCATS-CL4235**
Institution	**National Institutes of Health National Center for Advancing Translational Sciences Bethesda, Maryland, USA**
Contact information of distributor	**Dr. Wei Zheng,** wzheng@mail.nih.gov
Type of cell line	**iPSC**
Origin	**Human**
Additional origin info requiredfor human ESC or iPSC	**Age:9 months** **Sex: Male**
Cell Source	**Peripheral Blood**
Clonality	**Clonal**
Associated disease	**Alagille syndrome**
Gene/locus	p.Cys682Leufs*7 (c.2044dup) *JAG1* exon 16
Date archived/stock date	**2023**
Cell line repository/bank	**Coriell Institute of Medical Research** https://hpscreg.eu/cell-line/TRNDi032-A
Ethical approval	**Patients were consented into an IRB-approved study (92-000527) at the Children’s Hospital of Philadelphia and the study team obtained written consent.**

## 2. Resource utility

The study of ALGS pathophysiology may be enhanced via the utilization of this human-induced pluripotent stem (iPSC) as a cell-based model. Additionally, it is possible that hepatocytes may be derived from this cell line and be used in *in vitro* assays to further elucidate potential therapeutics for patients with ALGS.

## 3. Resource details

Alagille syndrome is a rare multifaceted disorder that occurs mostly due to mutations in the *JAG1* gene and rarely due to mutations in the *NOTCH2* gene. There are currently no early predictors for hepatic outcome in patients with ALGS. In addition to this, there are also no correlations between genotypic or phenotypic features and end stage liver disease in patients, which makes treatment especially difficult. 97% of cases are due to haploinsufficiency of the *JAG1* gene (Singh et al., 2018) with the most common mutation being on the 20p11.220p12 chromosome. On the other hand, only 2 % of cases are associated with *NOTCH2* mutations. Both of these genes are components of the Notch signaling pathway. ALGS patients usually exhibit paucity in intrahepatic bile ducts and the presence of three out of five other clinical criteria (cholestatic liver diseases, cardiovascular malformations, etc). Though maralixibat chloride (Livmarli) has been approved recently for reducing the symptom of itchy skin in ALGS patients, there is no known cure for ALGS and current treatment options are either highly invasive, or are limited to symptom management by multidisciplinary teams (Sanchez et al., 2021).

**Fig. 1.**
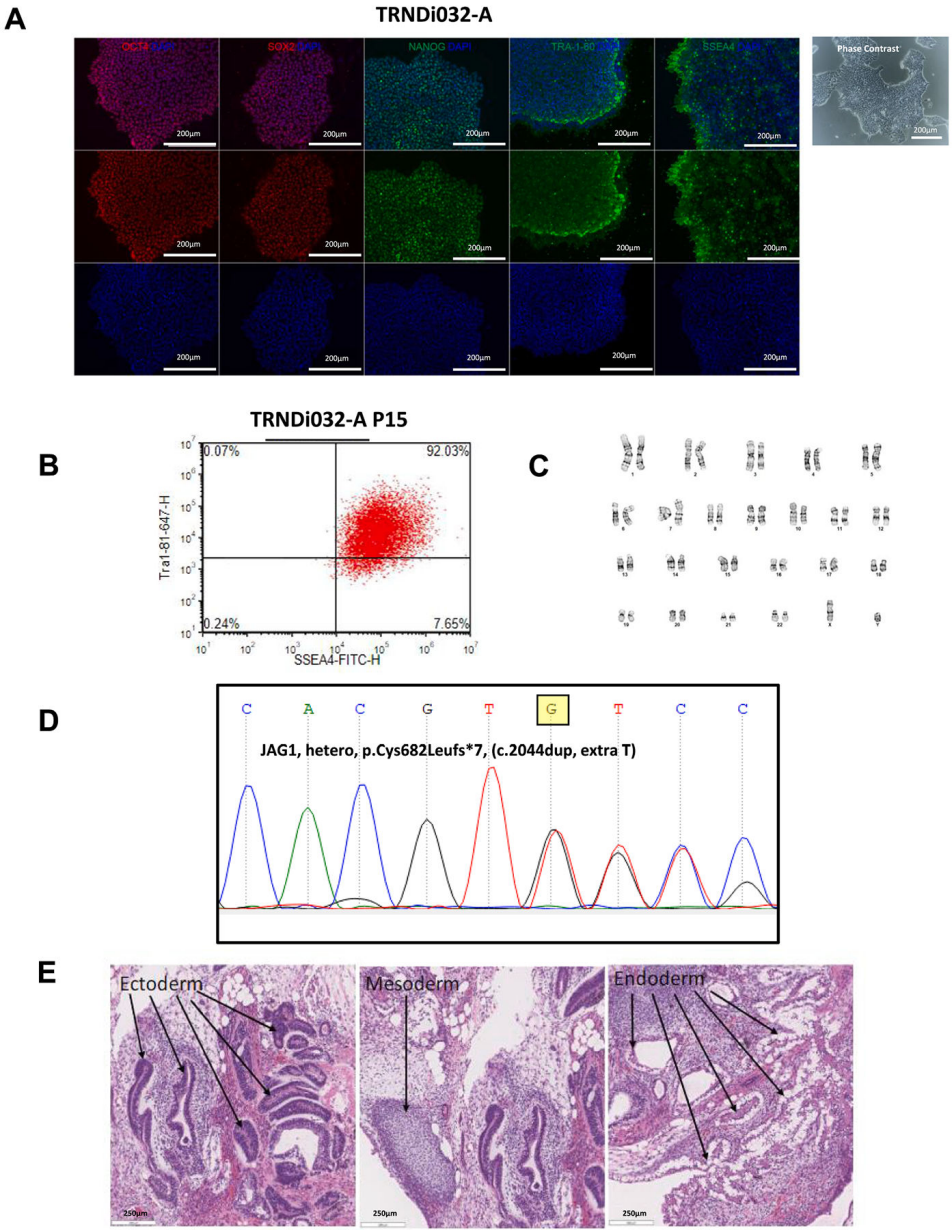
Characterization of TRNDi032-A iPSC line. (A) Left: Immunofluorescence images of iPSCs positive for stem cell markers: OCT4, SOX2, SSEA4, TRA-1-60, and NANOG. Nucleus is labeled with Hoechst 33342 (blue). Right: Phase contrast image of TRNDi032-A colonies. Scale bars represent 200 um. (B) Flow cytometry analysis of pluripotency protein markers: SSEA4, TRA-1-81. (C) Cytogenetic analysis showing a normal karyotype (46, XY). (D) Detection of heterozygous gene mutation of p.Cys682Leufs*7 (c.2044dup) in *JAG1* exon 16. (E) Analysis of teratoma from TRNDi032-A iPSC showing differentiation of three germ layers (ectodermal, mesodermal, and endodermal differentiation). Scale bars represent 250 um.

This study has established a human iPSC line (**TRNDi032-A**) from the lymphoblasts of a 9 month old male patient (Children’s Hospital of Philadelphia) carrying a heterozygous mutation, p.Cys682Leufs*7 (c.2044dup), which created a frameshift mutation in exon 16 of *JAG1* (Gilbert et al., 2019). The ALGS iPSC line was generated via episomal reprogramming, containing OCT4, KLF4, SOX2, and C-MYC pluripotency transcription factors. Colonies were selected, expanded, and then analyzed at the genetic and cellular level to validate successful reprogramming (Table 1). The iPSC line that was selected exhibited normal embryonic stem cell morphology under phase-contrast microscopy and expressed pluripotency markers OCT4, NANOG, SOX2, SSEA4 and TRA-1-60 (Fig. 1A). Flow cytometry showed co-expression of pluripotency marker TRA-1-81 and SSEA-4 in 92.03 % of cells, validating the presence of both markers (Fig. 1B). G-banded karyotype analysis showed a normal karyotype (46, XY) with no observed abnormalities (Fig. 1C). The expected genetic mutation was observed by whole genome sequencing (Fig. 1D). Pluripotency was verified by a teratoma formation experiment that confirmed the ability of the cell line to differentiate into three germ layers (ectoderm, mesoderm, endoderm) in vivo (Fig. 1E). The iPSC line tested negative for mycoplasma contamination (Fig. S1). Also, the STR DNA profile of this cell line matched with its parental cell line AGS591P (archived with journal).

## 4. Materials and methods

### 4.1. Cell culture and reprogramming

Patient peripheral blood cells were obtained from the Children’s Hospital of Philadelphia and were reprogrammed using episomal reprogramming technology. In terms of the reprogramming process, donor cells were centrifuged, resuspended, and then transferred to 1.5 ml Eppendorf tube with Epi5 reprogramming vectors (Thermo Cat#: A15960). Following this cells were transfected using the Neon Transfection System (Thermo # MPK5000). Once transfection was complete, cells were transferred to a 12-well plate with LCL medium (RPMI-1640, 15 % FBS, 1 % Glutamax) and allowed to recover overnight. Cells were then cultured in TeSR-E7 Medium (Stem Cell Technologies, #05914) for 16 days, and then subsequently in E8 Medium (Thermo #A1517001) for an additional 4 days. Clones were then picked on day 21. Regarding iPSC cell culturing, patient iPSCs were cultured in StemFlex media (Gibco, A33493-01) on 0.1 mg/mL Matrigel (Corning, 354277)-coated plates or Geltrex (A1413201) - coated plates at 37 °C in humidified air with 5 % CO2 and 5 % O2. The cells were passaged with EZ-LiFT^™^ (Sigma-Aldrich) at a generally 1:8 ratio when they reached 70 % confluency with 10 μM ROCK inhibitor.

### 4.2. Genome analysis

Sanger sequencing to confirm mutation was conducted after passage 10. Genomic DNA was purified from the iPSC line using AMPure XP at 1X ratio on the Biomek 4000 and premix sent out to sequencing according to the vendor guidelines. Target is amplified with Phusion^®^ Hot Start Flex DNA Polymerase 40 cycles Ta62. on the T100 Thermal Cycler from Bio-Rad (#1861096) using the following program: 98 °C, 30 s; 35 cycles of [98 °C, 10 s; 60 °C, 15 s; 72 °C, 30 s], 72 °C 5 min; 4 °C, indefinite. Genotyping for the variant was performed using whole genome sequencing. The specific primers for gene amplification and sequencing are listed in Table 2.

### 4.3. Immunocytochemistry

Patient iPSCs cultivated after passage 10 on a 96-well plate were fixed with 4 % paraformaldehyde for 15 min, at room temperature. After washing twice with DPBS, cells were permeabilized with 0.3 % Triton X-100 (Sigma) in DPBS for 15 min and followed by blocking buffer (Cell Staining Buffer, BioLegend) for 1 hr. The cells were then incubated with primary antibodies, diluted in the blocking buffer, overnight at 4 °C. Cells were washed twice with DPBS and incubated with secondary antibodies for 1 hr at room temperature (Table 2). Cell nuclei were stained with Hoechst 33,342 for 15 min and imaged with the Invitrogen EVOS M5000 (Thermo Fisher). Adobe Illustrator (Bethesda, MD, NIH) was used to produce the image montage.

### 4.4. Flow Cytometry analysis

After clone selection cells were expanded, cells were dissociated, washed once with DPBS and then spun for 5 min at 200 g. After spin, supernatant was discarded, and cells were resuspended for staining in 100 μl DPBS buffer containing 2 % FBS. Cells were then divided into two separate Eppendorf tubes with 100 μl of cells in each one. Afterwards, staining was done with fluorophore-conjugated antibodies (Table 2) for 20 min at 4 °C. Cells were resuspended in 250 μl PBS wash and then analyzed with a BD Accuri C6 Flowcytometry system (BD Biosciences).

### 4.5. G-banding karyotype (change)

The G-banded karyotyping analysis was performed after passage 10 by Cell Line Genetics, Inc. Twenty randomly selected metaphase cells were used for the standard cytogenetic analysis.

### 4.6. Short tandem repeat (STR) DNA profile analysis (change)

STR analyses of patient fibroblasts and derived iPSCs after passage 10 were performed by Cell Line Genetics, Inc. for STR analysis (Cell Line Genetics, #STR- 100). The genomic DNA of parental LCLs and derived iPSC were prepared using Qiagen DNeasy Blood & Tissue Kits (Qiagen # 69506). GenePrint^®^ 24 System was used for co-amplification with a five-dye profile of 23 STR loci and Amelogenin.

### 4.7. Mycoplasma detection

Cell line media was first spun at high speed for 20 min, then supernatant was discarded. Afterwards, 50 μl of Phusion^®^ HF Buffer from NEB (B0518S) at 0.5x with Proteinase K at 8U/ml (NEB P8107S) was added. Lysis occurred at 55 °C for 1–3 h. Proteinase K was inactivated via heat at 95 °C for 10 min. 2 μl of lysis was used as a template with a GPO1/MGSO mycoplasma specific primer pair, and endogenous GAPDH primers in qPCR amplification with Bio-Rad SsoFast EvaGreen Supermix.

### 4.8. Teratoma formation assay

After passage 10 Patient iPSCs were dissociated with EZ-LiFT^™^ and resuspended approximately 1 × 10^7^ cells in 400 μl culture medium supplemented with 10 mM HEPES (pH 7.4). Afterward, 200 μl cold Matrigel (Corning, 354277) was mixed with the cells. The cell suspension was injected subcutaneously into NSG mice (JAX No. 005557) at 150 μl per injection site. Visible tumors were harvested 6–8 weeks postinjection and immediately fixed in 10 % Neutral Buffered Formalin. The fixed tumors were then embedded in paraffin, sliced, and stained with hematoxylin and eosin.

## Supplementary Material

MMC1

## Figures and Tables

**Table 1:** Characterization and validation.

Classification	Test	Result	Data
**Morphology**	**Photography Bright field**	**Normal**	Fig. 1 **Panel A**
**Phenotype**	**Immunocytochemistry**	**SOX2, OCT4, TRA-1-60, NANOG, SSEA-4**	Fig. 1 **Panel A**
	**Flow cytometry**	**Co-staining of TRA-1-81 and SSEA-4 (92.03 %)**	Fig. 1 **Panel B**
**Genotype**	**Karyotype (G-banding) and resolution**	**46 XYResolution 400–425 bp**	Fig. 1 **Panel C**
**Identity**	**Microsatellite PCR (mPCR) OR**	**n/a**	**n/a**
	**STR analysis**	**16 sites tested; all sites matched**	**Submitted in an archive with journal**
**Mutation analysis (IF APPLICABLE)**	**Whole Genome Sequencing**	Heterozygous mutation, *JAG1,* p.Cys682Leufs*7 (c.2044dup)	Fig. 1 **Panel D**
	**Southern Blot OR WGS**	**N/A**	**N/A**
**Microbiology and virology**	**Mycoplasma**	**Mycoplasma testing by luminescence. Negative**	Supplemental Fig. 1
**Differentiation potential**	**Teratoma formation**	**Teratoma with three germ layers formation. Ectoderm (neural epithelium); Mesoderm (cartilage); ectoderm (gut-like tissue)**	Fig. 1 **Panel E**
**List of recommended germ layer markers**	**Expression of these markers has to be demonstrated at mRNA (RT PCR) or protein (IF) levels, at least 2 markers need to be shown per germ layer**	*N/A*	*N/A*
**Donor screening (OPTIONAL)**	HIV 1 + 2 Hepatitis B, Hepatitis C	**N/A**	**N/A**
**Genotype additional info (OPTIONAL)**	Blood group genotyping	**N/A**	**N/A**
	HLA tissue typing	**N/A**	**N/A**

**Table 2:** Reagents details.

	Antibodies used for immunocytochemistry/flow-cytometry			
	Antibody	Dilution	Company Cat #	RRID
Pluripotency Markers	**Mouse anti-SOX2**	***1:200***	**R & D systems, Cat# MAB2018**	**RRID: AB_358009**
Pluripotency Markers	**Goat anti-OCT3/4**	***1:200***	**R & D, Cat# AF1759**	**RRID: AB_354975**
Pluripotency Markers	**Mouse anti-TRA-1-60**	***1:200***	**Cell signaling, Cat# 4746**	**RRID: AB_2119059**
Pluripotency Markers	**Rabbit anti-NANOG**	**1:200**	**Cell signaling, Cat# 4903**	**RRID: AB_10559205**
Pluripotency Markers	**Mouse anti-SSEA4**	**1:200**	**Cell signaling, Cat# 4755**	**RRID: AB_1264259**
Secondary Antibodies	**anti-TRA160 Donkey Anti-Mouse IgM (Alexa Fluor 488)**	**1:1000**	**Jackson Immunoresearch, Cat# 715-545-140**	**RRID: AB_2340845**
Secondary Antibodies	**anti-NANOG Donkey Anti-Rabbit IgG (H + L) (Alexa Fluor 488)**	**1:1000**	**Jackson Immunoresearch, Cat# 711-545-152**	**RRID: AB_2313584**
Secondary Antibodies	**anti-SOX2 Donkey Anti-Mouse IgG (H + L) (Alexa Fluor 594)**	**1:1000**	**Jackson Immunoresearch, Cat#715-585-151**	**RRID: AB_2340855**
Secondary Antibodies	**anti-OCT3/4 Donkey Anti-Goat IgG (H + L) (Alexa Fluor 594)**	**1:1000**	**Jackson Immunoresearch, Cat#705-585-147**	**RRID: AB_2340433**
Secondary Antibodies	**anti-SSEA4: F(ab’)2-Goat anti-Mouse IgG (H + L) (Alexa Fluor 488)**	**1:1000**	**Thermo Fisher Cat#A-11017**	**RRID: AB_2534084**
Flow Cytometry Antibodies	**FITC Mouse anti-SSEA-4**	**1:200**	**BD Pharmingen^™^ Cat# 560126**	**RRID: AB_1645491**
Flow Cytometry Antibodies	**FITC Mouse IgG3, Isotype Control**	**1:200**	**BD Pharmingen^™^ Cat# 555578**	**RRID: AB_395956**
Flow Cytometry Antibodies	**anti-Human TRA-1-81 Antigen**	**1:500**	**Alexa Fluor^®^ Cat# 560124**	**RRID: AB_1645449**
Flow Cytometry Antibodies	**Mouse IgM, Isotype Control**	**1:500**	**Alexa Fluor^®^ 647 Cat# 560806**	**RRID: AB_2034030**
Flow Cytometry Antibodies	**Anti-SSEA-4-BD Pharmingen**	**1:10**	**BD, Cat# 560126**	**RRID: AB_10807973**
	**Primers**			
	**Target**	**Size of band**	**Forward/Reverse primer (5′-3′)**	
**Targeted Mutation analysis (PCR)**	***JAG1***	**783 bp**	**CCACCCCACATCTTGCCTAA/ AGACGCACGTAAAGGACTCG**	
	**GAPDH**	**1231 bp**	**GGGAGCCAAAAGGGTCATCA/TGATGGCATGGACTGTGGTC**	
	**GPO-1**		**ACGGCCCAGACTCCTACGGGAGGCAGCAGTA**	
	**MGSO**		**CCATGCACCATCTGTCACTCTGTTAACCTC**	

## Data Availability

STR data is archived with journal
