# Supplementary material for: Generation of an Alagille syndrome (ALGS) patient-derived induced pluripotent stem cell line (TRNDi032-A) carrying a heterozygous mutation (p.Cys682Leufs*7) in the JAG1 gene
Source: Stem Cell Res. Author manuscript; Available in PMC 2024 Feb 5. (PMC10842201; doi:10.1016/j.scr.2023.103231)
Supplement: MMC1 [file NIHMS1943036-supplement-MMC1.docx]

**Supplementary Figure S1**. Mycoplasma detection performed on TRNDi032-A iPSCs

| **Primer** | **Control (Pos/Neg)** | **Cell Line** |  |  |
| --- | --- | --- | --- | --- |
| GPO-1_MGSO | N/A | HT_977Ap15 | -2.6 |  |
| GPO-1_MGSO | N/A | HT_977Bp15 | -1.64 |  |
| GPO-1_MGSO | Neg Ctrl | MM1_-CTL | 54.17 |  |
| GPO-1_MGSO | Pos Ctrl | MM2_+CTL | 16246.4 |  |
| GAPDH-3 | Unkn | HT_977Ap15 | 14121.03 |  |
| GAPDH-3 | Unkn | HT_977Bp15 | 15027.9 |  |
| GAPDH-3 | Neg Ctrl | MM1_-CTL | 52.0 |  |
| GAPDH-3 | Pos Ctr | MM2_+CTL | 14162.37 |  |
